# The differential impact of intraventricular and interventricular dyssynchrony on left ventricular remodeling and function in patients with isolated left bundle branch block

**DOI:** 10.1186/1532-429X-14-S1-P149

**Published:** 2012-02-01

**Authors:** Valentina Valenti, Mohammad I Zia, Azhar Supariwala, Leon Shubayev, Sophia Edelstein, Matt Levin, Seth Uretsky, Steven D Wolff

**Affiliations:** 1 Advanced Cardiovascular Imaging, New York, NY, USA; 2 Radiology, University "La Sapienza," Sant'Andrea Hospital, Rome, Italy; 3 Medicine, St. Luke's Roosevelt Hospital Centre, New York, NY, USA; 4 Sunnybrook Health Sciences Centre, Toronto, ON, Canada

## Summary

Interventricular dyssynchrony evaluated with cardiac magnetic resonance is an independent predictor of systolic dysfunction and left ventricular remodeling while intraventricular dyssynchrony is an independent predictor of LV diastolic dysfunction in patients with isolated LBBB. Comprehensive evaluation of both these parameters of dyssynchrony would be useful in selecting patients for cardiac resynchronization therapy.

## Background

Dyssynchrony in patients with left bundle branch block (LBBB) plays an important role in the development of left ventricular (LV) dilation, systolic dysfunction, progression of heart failure and mortality. Intraventricular dyssynchrony (IntraVD) and interventricular dyssynchrony (InterVD) may have different impacts on myocardial structure and function in patients with LBBB. Our objective was to characterize the independent effects of IntraVD and InterVD on myocardial structure and performance.

## Methods

Thirty-two patients with isolated LBBB (15 males, mean age: 61 years ± 13) were assessed using cardiac magnetic resonance imaging. IntraVD was defined as the difference between the time of maximum systolic wall thickness of the septum and lateral wall of the left ventricle. InterVD was defined as the time difference between the onset of pulmonic and aortic flow. Peak filling rate (PFR) and time to peak filling rate (TPFR) were measured as surrogates of diastolic function.

## Results

InterVD had a moderate inverse negative correlation with LV ejection fraction (r2=-0.5), and a moderate positive correlation with LV end-diastolic volume index (LVEDVI) (r2=0.4) and LV end-systolic volume index (LVESVI) (r2=0.5). The IntraVD had no significant correlation with LV remodeling and function parameters. Multivariate analysis also demonstrated that InterVD was an independent predictor of left ventricular systolic dysfunction (p<0.0001), increased LVEDVI (p<0.01), and increased LVESVI (P<0.001).

In terms of diastolic dysfunction, IntraVD had a moderate positive correlation with THFR (r2= 0.5) and moderate negative correlation with PFR (r2=-0.5). InterVD was not significantly correlated with any diastolic dysfunction parameter. Multivariate analysis confirmed that IntraVD was an independent predictor of left ventricular diastolic dysfunction (PFR: p< 0.001; TPFR: p<0001).

## Conclusions

InterVD evaluated with CMR is an independent predictor of systolic dysfunction and left ventricular remodeling. IntraVD evaluated with CMR is an independent predictor of LV diastolic dysfunction. A comprehensive evaluation of both these independent measures of dyssynchrony could be helpful in improving the patient selection algorithm for cardiac resynchronization therapy.

## Funding

None.

